# The serine protease, dipeptidyl peptidase IV as a myokine: dietary protein and exercise mimetics as a stimulus for transcription and release

**DOI:** 10.14814/phy2.12827

**Published:** 2016-06-22

**Authors:** Leslie E. Neidert, C. Brooks Mobley, Wesley C. Kephart, Michael D. Roberts, Heidi A. Kluess

**Affiliations:** ^1^ School of KinesiologyAuburn University301 Wire RoadAuburnAlabama36849

**Keywords:** DPP‐IV, exercise, whey protein

## Abstract

Dipeptidyl‐peptidase IV (DPP‐IV) is an enzyme with numerous roles within the body, mostly related to regulating energy metabolism. DPP‐IV is also a myokine, but the stimulus for its release is poorly understood. We investigated the transcription and release of DPP‐IV from skeletal muscle in a three‐part study using C_2_C_12_ myotube cultures, an acute rat exercise and postexercise feeding model, and human feeding or human exercise models. When myotubes were presented with leucine only, hydrolyzed whey protein, or chemicals that cause exercise‐related signaling to occur in cell culture, all caused an increase in the mRNA expression of DPP‐IV (1.63 to 18.56 fold change, *P* < 0.05), but only whey protein caused a significant increase in DPP‐IV activity in the cell culture media. When rats were fed whey protein concentrate immediately following stimulated muscle contractions, DPP‐IV mRNA in both the exercised and nonexercised gastrocnemius muscles significantly increased 2.5‐ to 3.7‐fold (*P* < 0.05) 3–6 h following the exercise/feeding bout; of note exercise alone or postexercise leucine‐only feeding had no significant effect. In humans, plasma and serum DPP‐IV activities were not altered by the ingestion of whey protein up to 1 h post consumption, after a 10 min bout of vigorous running, or during the completion of three repeated lower body resistance exercise bouts. Our cell culture and rodent data suggest that whey protein increases DPP‐IV mRNA expression and secretion from muscle cells. However, our human data suggest that DPP‐IV is not elevated in the bloodstream following acute whey protein ingestion or exercise.

## Introduction

Dipeptidyl peptidase‐IV (DPP‐IV) is a serine protease that is present in the gut, kidneys, endothelial layer of blood vessels and as a soluble protein in the blood. Pleiotropic functions of DPP‐IV include modulation of the function of glucagon like peptide‐1 (GLP‐1), neuropeptide Y, and peptide YY; all of which play an important role in insulin release and satiety (Badman and Flier 2005; Batterham et al. 2006; Loh et al. 2015). DPP‐IV is also expressed on the surface of T‐cells as the antigen CD26+, which activates CD4+ cells to release tumor necrosis factor‐*α* (TNF‐*α*) and interleukin‐6 (IL‐6) to induce T‐cell proliferation and initiate an immune response (Cordero et al. 2009; Ikeda et al. 2013). Recent work suggests that DPP‐IV may also be released as a myokine from human myotubes (Raschke et al. 2013), but the pathway for release of DPP‐IV and the function of DPP‐IV once released from the muscle remain unknown.

It is reported that whey protein may be a potent DPP‐IV inhibitor. Evidence for this action of whey protein comes from studies using whey protein or its products in an assay with recombinant DPP‐IV (Tulipano et al. 2011; Silveira et al. 2013) and studies with humans or animals ingesting whey protein and measuring an increase in GLP‐1 or insulin levels (Frid et al. 2005; Salehi et al. 2012). However, it remains unknown if the inhibitory effect of whey protein on DPP‐IV activity modulates other aspects of DPP‐IV production (i.e., transcription, secretion, etc.). Moreover, it remains unknown if amino acids themselves affect aspects of DPP‐IV production or if this effect is confined to intact whey protein.

Therefore, the purpose of this study was twofold: (1) to identify if leucine, whey protein and/or exercise affected various aspects of DPP‐IV production in skeletal muscle, and (2) to determine if whey protein has an inhibitory effect on DPP‐IV secretion from skeletal muscle. Our experimental approach employed C_2_C_12_ myotube cultures, an acute rat exercise and postexercise feeding model, and human feeding or human exercise models. We hypothesized that aspects of DPP‐IV production as well as release by the muscle would be potentiated by muscle contractions. Also, given the aforementioned data regarding the ability of whey protein to stimulate increases in circulating insulin and GLP‐1, we hypothesized that whey protein would inhibit DPP‐IV mRNA expression and DPP‐IV release from muscle.

## Materials and Methods

### Ethical approval

All animal and human experiments were approved by the Auburn University Institutional Animal Care and Use Committee and the Institutional Review Board, respectively, prior to the beginning of any data collection.

### Part 1: Modulation of DPP‐IV in cell culture using exercise‐related signaling pathways, leucine and whey protein

#### Cell culture and treatments

C_2_C_12_ myoblasts were grown on 60 mm plates (Griener Bio‐One GmbH, Maybachstr, Frickenhausen, GER) in growth medium [GM; Dulbecco's Modified Eagle Medium (DMEM), 10% (vol/vol) fetal bovine serum, 1% penicillin/streptomycin, 0.1% gentamycin] at a seeding density of 3.5 × 10^5^ at 37°C in a 5% CO_2_ atmosphere. Forty‐eight hours after myoblast growth reached 80–90% confluency, differentiation was induced by replacing the GM with differentiation medium [DM; DMEM, 2% (vol/vol) horse serum, 1% penicillin/streptomycin, 0.1% gentamycin]. For 7 days following differentiation induction, DM was replaced every 24 h to allow for myotube growth.

Following the 7 days of differentiation, cells were treated on a single occasion for 6 h with: (1) DMEM only (CTL, *n* = 6), (2) 10 mmol/L (1.3 *μ*g/mL) l‐leucine (LEU, *n* = 6) (EMD Chemicals, Inc.), (3) 100 mmol/L (13 *μ*g/mL) whey protein hydrolysate (WP, *n* = 6) (MusclePharm, Corp., Denver, CO), (4) 5 mmol/L (1.0 *μ*g/mL) Caffeine (CAFF, *n* = 6) (Ameresco, Pelham, AL), (5) 1 mmol/L (0.012 *μ*L/mL) H_2_O_2_ (*n* = 6) (Ameresco), and 6) 2 mmol/L (0.5 *μ*g/mL) 5‐Aminoimidazole‐4‐carboxamide ribonucleotide (AICAR, *n* = 6) (Ameresco). DMEM served as the vehicle for each respective treatment. The LEU concentration was based on a study showing that respective concentration amplified Akt phosphorylation (p) in C_2_C_12_ myotubes (DeLong et al. 2011). The WP concentration was based upon the amino acid profile of WP, for which LEU constitutes ~12–14% of the amino acid content (Hulmi et al. 2010).

In addition to the effects of LEU and WP, we were also interested in examining if “exercise‐like” signals promoted changes in DPP‐IV mRNA levels or DPP‐IV activity in the culture media. Previous studies have reported that, during resistance exercise of skeletal muscle, increases in intramuscular calcium (Beaton et al. 2002), reactive oxygen species (Reid and Durham 2002), and AMPK (Dreyer et al. 2006) activation occur. The CAFF dosage was based on a study showing a 5 mmol/L concentration was able to increase intracellular calcium levels in L6 myotubes (Barres et al. 2012). The AICAR dosage was based on a previous study that reported an increase in the activation of AMPK in C_2_C_12_ myotubes at the 2 mmol/L concentration (Egawa et al. 2014). The H_2_O_2_ dosage was supported by a study that showed increased atrophy‐related mechanisms at this dosage (McClung et al. 2009).

A post hoc set of cell culture experiments were performed investigating the role of metalloproteases in the process of shedding DPP‐IV from the cell membrane of skeletal muscle cells. This mechanism was previously proposed as the mechanism by which DPP‐IV is released from various cell types (Lamers et al. 2011; Röhrborn et al. 2014). To this purpose we used inhibition of metalloproteases (MMP) 2 and 9 in combination with whey protein or leucine, based on evidence of these MMPs being involved in the shedding of DPP‐IV from muscle cells (Röhrborn et al. 2014). Following the same procedures as above, nine plates of C_2_C_12_ cells were treated for 6 h with: (1) 12 nmol/L MMP2 inhibitor (MMP2i; dissolved in DMSO according to manufacturer's instructions; EMD Biosciences, San Diego, CA), (2) MMP2i + WP, (3) MMP2i + LEU, (4) 5 nmol/L MMP9 inhibitor (MMP9i; dissolved in DMSO according to manufacturer's instructions; EMD Biosciences, San Diego, CA), (5) MMP9i + WP, (6) MMP9i + LEU, (7) Complete protease inhibitor (CPI; dissolved in DMEM according to manufacturer's instructions; Roche Diagnostics Corporation, Indianapolis, IN) (8) CPI + WP, and (9) CPI + LEU.

#### DPP‐IV mRNA

The production of mRNA was measured by real‐time PCR of the myotubes. Briefly, myotubes were lysed using 500 *μ*L of Ribozol (Ameresco) per the manufacturer's recommendations. Total RNA concentrations were analyzed using a Nanodrop Lite spectrophotometer (Thermo Scientific, Waltman, MA), and 2 *μ*g of cDNA was synthesized using a commercial qScript^™^ cDNA SuperMix (Quanta Biosciences, Gaithersburg, MD) as recommended by the manufacturer. Real‐time PCR was performed using gene‐specific primers and SYBR green chemistry. The primer sequences were as follows: DPP‐IV forward (5′→3′): CAGCTCATCCTCTAGTGCGG, DPP‐IV reverse (5′→3′): TCTTGCCACAGATGCAGGAG, Gusb (housekeeping gene) forward (5′→3′): TCAGCTCTGTGACCGATACG, Gusb reverse (5′→3′): GCCACAGACCACATCACAAC, Rer1 (housekeeping gene) forward (5′→3′): GCCTTGGGAATTTACCACCT, Rer1 reverse (5′→3′): CTTCGAATGAAGGGACGAAA, Rpl‐7l1 (housekeeping gene) forward (5′→3′): ACGGTGGAGCCTTATGTGAC, Rpl‐7l1 reverse (5′→3′): TCCGTCAGAGGGACTGTCTT. Fold‐change values from the respective control treatment were performed using the Livak method (i.e., 2^−ΔΔCT^ assuming 100% primer binding efficiency), where 2^−ΔCT^ = [housekeeping gene (HKG) critical threshold (CT) – gene of interest CT] and 2^−ΔΔCT^ (or fold‐change) = [2^−ΔCT^ value/2^−ΔCT^ average of respective control treatment]. Beta‐glucuronidase (Gusb) was used as a HKG for all “nutrient signal” comparisons given that it remained stable across all treatments (CTL PCR Critical threshold mean ± standard error: 25.63 ± 0.09; LEU: 25.86 ± 0.09; WP: 25.21 ± 0.09; ANOVA *P* > 0.10). The geometric mean of retention in endoplasmic reticulum receptor 1 (Rer1) and ribosomal protein 7 like 1 (rpl‐71l) was used as the HKG values for all “exercise‐like” treatment comparisons given that it remained stable across all treatments (CTL PCR Critical threshold mean ± standard error: 32.88 ± 0.29; CAFF: 33.14 ± 0.08; H_2_O_2_: 32.55 ± 0.15; AICAR: 33.22 ± 0.16; ANOVA *P* > 0.07).

#### DPP‐IV activity

Samples of the culture media were taken 6 h after the treatments. The DPP‐IV activity in the media was determined using a fluorometric assay developed by Scharpé et al. (Scharpé et al. 1988), as used previously by our laboratory (Evanson et al. 2011) for homogenates and by other laboratories for cell culture media (Dourado et al. 2007). A modification to the protocol included the use of cell culture media in place of Kreb's Ringer Warm buffer for the Sample Blank. DPP‐IV activity was determined by the following equation:

$$\text{Activity (U/L)}=\left[\left(S\right)\times V_{A}\times 1000\times C_{\mathrm{st}}\right)]/\left[\left(T\times S_{v}\right)\times \left(F\right)\right]$$

where *S* is the sample fluorescence minus the sample blank fluorescence, *V*_*A*_ is the total volume of the well, *C*_st_ is the standard concentration, *T* is the time of incubation, *S*_*v*_ is the sample volume, and *F* is the standard fluorescence minus the standard blank fluorescence. Matheeussen et al. (2012) determined the detection limit of the assay to be 0.1 U/L and the between‐run variation coefficient range to be 1.32–3.32%. Additionally, the specificity for DPP‐IV was supported when 98% of activity was inhibited by addition of a DPP‐IV selective inhibitor to samples.

### Part 2: Modulation of DPP‐IV in animal model with muscle stimulation, amino acid feeding and whey protein feeding

#### Animal supplementation and exercise protocol

Male Wistar rats (~250 g; Harlan Laboratories, Indianapolis, IN) were acclimated 5 days prior to experimentation in the campus animal housing facility. Animal quarters were kept at ambient room temperature on a constant 12 h light: 12 h dark cycle. Water and standard rodent chow (24% protein, 58% carbohydrate, 18% fat; Teklad Global #2018 Diet, Harlan Laboratories) were provided to animals ad libitum.

Beginning the day prior to the acute exercise and feeding experiment, animals underwent an 18 h overnight fast following removal of food from the home cages. The morning of experimentation, animals were removed from their quarters between 0700 and 0800, transported to the School of Kinesiology building, and acclimated for approximately 3–4 h. After acclimation, rats were anesthetized using isoflurane and then resistance trained via electrical stimulation producing dynamic plantarflexion movements. This muscle stimulation procedure is described elsewhere (Mobley et al. 2016). Briefly, animals were fastened to an apparatus that allowed the two hindlimbs to move freely. Two subcutaneous electrodes connected to a Grass S48 Stimulator (Grass Medical Instruments, Quincy, MS) were placed parallel to the gastrocnemius in each rat's right leg. Four sets of eight stimulations (70 mV, 100 Hz, 2s train duration, 0.2 TPS train rat, and 0.2 ms duration) were delivered, with 2 min of recovery between sets. Immediately following the final training set, rats were administered either 500 mg of whey protein (MusclePharm, Denver, CO; which is a proprietary blend prominently comprised of WP concentrate; *n* = 10 rats), 54 mg of LEU (EMD Chemicals, Inc., San Diego, CA; *n* = 8 rats), or 1 mL of water (CTL; *n* = 10 rats). The species conversion calculations of Reagan‐Shaw et al. (2008) was used to determine whey protein dosage, where the human body mass for an average male was assumed to be 80 kg. This resulted in rats receiving approximately an 18.8 g human equivalent dose of WP. The LEU dose was based on the same concept as mentioned in Part 1. The resulting dosage that LEU‐treated rats received was approximately a 2.8 g human equivalent dose. Each supplement was suspended in 1 mL of water and administered via gavage feeding. Animals were kept under light isoflurane anesthesia postexercise for approximately 30 s while gavage feeding occurred. Following the exercise and feeding protocol, rats were allowed to recover 3–6 h prior to being euthanized under CO_2_ gas in a 2 L induction chamber (VetEquip, Inc., Pleasanton, CA). Due to limited resources, water‐fed CTL rats were only killed at 3 h post exercise and not 6 h post exercise. Moreover, blood was not extracted from these rats and, thus, serum DPP‐IV analyses were not performed. Of note, the “human relevancy” of this rodent feeding model has been validated elsewhere as Rasmussen and colleagues have shown the MPS response of humans and rats is similar following protein feedings (Butteiger et al. 2013; Reidy et al. 2013). Additionally, we have previously demonstrated that this exercise model increases muscle protein synthesis (Mobley et al. 2016).

#### Tissue preparation methods

Following euthanasia, approximately 50 mg of gastrocnemius muscle was extracted from the EX leg and the non‐EX leg and placed in 500 *μ*L of ice‐cold cell lysis buffer [20 mmol/L Tris‐HCl (pH 7.5), 150 mmol/L NaCl, 1 mmol/L Na‐EDTA, 1 mmol/L EGTA, 1% Triton, 20 mmol/L sodium Pyrophosphate, 25 mmol/L Sodium Fluoride, 1 mmol/L *β*‐glycerophosphate, 1 mmol/L Na_3_O4, 1 *μ*g/mL leupeptin]. Samples were homogenized via micropestle manipulation, and insoluble proteins from RIPA homogenates were removed by centrifugation at 500*g* for 5 min, and supernatants were assayed for total protein content using a BCA Protein Assay Kit (Thermos Scientific, Waltham, MA) prior to DPP‐IV activity analysis. Another 50 mg of gastrocnemius muscle was extracted from the EX leg and the non‐EX leg and placed in 500 *μ*L of Ribozol (Ameresco). Samples were then homogenized via micropestle manipulation and RNA isolation was performed per manufacturer's recommendations. Following RNA isolation, total RNA concentrations were analyzed using a Nanodrop Lite spectrophotometer (Thermo Scientific) and 2 *μ*g of gastrocnemius RNA was reverse transcribed into cDNA for real‐time PCR (RT‐PCR) analyses using a commercial qScript^™^ cDNA SuperMix (Quanta Biosciences, Gaithersburg, MD). RT‐PCR was performed using gene‐specific primers and SYBR‐green‐based methods in a RT‐PCR thermal cycler (3 min DNA polymerase activation at 95°C followed by 40 cycles of 95°C at 15 s and 60°C at 15 s) (Bio‐Rad Laboratories, Hercules, CA). Primers were designed using primer designer software (Primer3Plus, Cambridge, MA), and melt curve analyses demonstrated that one PCR product was amplified per reaction. The primer sequences were as follows: DPP‐IV forward (5′→3′): CACTCCACTGTTCCCCTCAC, DPP‐IV reverse (5′→3′): CTTGCAGTGGAAGAGCAGGA, rps16 (housekeeping gene) forward (5′→3′): TCGCTGCGAATCCAAGAAGT, rps16 reverse (5′→3′): CCCTGATCCTTGAGACTGGC. Past primer efficiency curves generated from our laboratory using this method of primer design and PCR protocol have yielded primer efficiencies within 90–110% (McGinnis et al. 2015; Mobley et al. 2015). Fold‐change values from the CTL non‐EX condition were performed using the Livak method (i.e., 2^−ΔΔCT^ assuming 100% primer binding efficiency), where 2^−ΔCT^ = [housekeeping gene (HKG) CT – gene of interest CT] and 2^−ΔΔCT^ (or fold‐change) = [2^−ΔCT^ value/2^−ΔCT^ average of CTL non‐EX condition]. Of note, for analyses in vivo*,* ribosomal protein S16 (Rps16) was used as a HKG given that it remained stable across all treatments and EX conditions (Ctl‐NX PCR critical threshold (Ct) mean ± standard error: 20.55 ± 0.11; Ctl‐EX: 20.43 ± 0.11; 3 h Leu NX: 20.31 ± 0.12; 3 h Leu‐EX: 20.42 ± 0.12; 6 h Leu‐NX: 20.62 ± 0.11; 6 h Leu‐EX: 20.41 ± 0.08; 3 h WP‐NX: 20.48 ± 0.10; 3 h WP‐EX: 20.18 ± 0.05; 6 h WP‐NX: 20.75 ± 0.17; 6 h WP‐EX: 20.32 ± 0.15; ANOVA *P* > 0.10).

#### DPP‐IV activity

The DPP‐IV activity of muscle homogenate was determined using the same DPP‐IV assay as described in Part 1, excluding the modification of the use of cell culture media. This assay has been used previously in other studies to measure the DPP‐IV activity of homogenates (Zhang et al. 2010; Evanson et al. 2011).

### Part 3: Modulation of DPP‐IV in humans with whey protein feeding or different types of exercise

All participants were recruited from Auburn University to participate in each of the following studies. Each study consisted of a different set of individuals participating in the study. A written informed consent, health questionnaire, and PAR‐Q (if applicable) were obtained prior to any data collection.

#### Whey protein

Nineteen college aged males and females (age range = 19‐35; males *n* = 8, females *n* = 11) came into the laboratory after an overnight fast and consumed a standard commercially available whey protein shake (packet of 31 g of protein; MusclePharm, Denver, CO). Capillary samples were collected immediately prior to consumption of the shake, 30 min post consumption, and 60 min post consumption. Pilot data from the Kluess laboratory demonstrated that samples taken via capillary draw have comparable results to samples taken via venipuncture (average coefficient of variation was <10%; data not shown). Samples were centrifuged, and the plasma was drawn off and frozen at −80°C until analyzed for DPP‐IV activity as described in Part 1.

#### Treadmill exercise

Thirteen college aged males and females (age range = 19–35; males *n* = 7, females *n* = 6) completed a 10‐min bout of vigorous running. Participants reported to the laboratory after an overnight fast and ran at a self‐selected speed >5.5 mph for 10 min. Capillary samples were obtained immediately prior to exercise and 10 min post exercise. Plasma was obtained by centrifuging the samples to separate the blood, then immediately freezing the drawn off plasma at −80°C until DPP‐IV activity analysis as described in Part 1.

#### Repeated resistance exercise bouts

Fifteen college aged males (age = 22 ± 2 years, age range = 19‐35, body mass = 87.9 ± 11.1 kg, BMI = 28.1 ± 3) completed a muscle damage resistance exercise protocol. A pretest visit occurred 1 week before the start of the muscle damage protocol. Baseline blood measures were collected via venipuncture in addition to the determination of the participant's one repetition maximum (1‐RM). Approximately 1 week following baseline testing, participants engaged in a resistance exercise bout that consisted of 10 sets of five free‐weighted back squats at 85% of the participant's 1‐RM for three consecutive days. If a participant could not complete the set of five, his weight was reduced until he could successfully complete the protocol. Participants completed this protocol over a three‐consecutive day period; the purpose of this protocol was to induce substantial muscle damage which was monitored through daily visual analog soreness scoring prior to and 48 h following the third lifting bout.

Blood was drawn prior to the 3‐day lifting protocol, and subsequent blood draws were obtained: (1) 24 h following bout 1, (2) 24 h following bout 2, and (3) 48 h following bout 3. All blood was collected from forearm veins using standard phlebotomy techniques and collected into 6 mL serum separator tubes. Serum was isolated by centrifuging blood tubes at 3500*g* for 5 min at room temperature, and was aliquoted in 1.7 mL tubes and stored at −80°C until batch DPP‐IV activity analyses as described in Part 1.

### Statistical analysis

Results of mRNA expression are presented as fold change from the control condition (CTL, MMP2i, MMP9i, or CPI). Results of DPP‐IV activity for cell culture media, serum, and plasma are presented as DPP‐IV activity per liter of sample, whereas the DPP‐IV activity in muscle homogenate is presented as DPP‐IV activity per milligram of protein. All results are reported as mean ± standard deviations. In order to determine significant differences in the DPP‐IV activity of the media and the DPP‐IV gene expression of the cells for the different treatments of Part 1, a one‐way ANOVA was used for both measures. For Part 2, a 3 × 3 × 2 ANOVA was used to determine the statistically significant changes of DPP‐IV mRNA expression and activity of the nonexercised and exercised legs with the different supplements at the different time points. In Part 3, a repeated measures ANOVA was used to determine significant differences in the activity of DPP‐IV among the different time points of the whey protein and muscle damage protocols, and a Student's *t*‐test was performed to determine the differences in DPP‐IV activity between the two time points of the vigorous running bout. Alpha was set at *P* < 0.05 to determine statistical significance of the results. If significant results of an ANOVA were found, a post hoc analysis was performed. All analyses were performed using GraphPad Prism 5 (GraphPad Software, Inc., La Jolla, CA) and IBM SPSS Statistics 22 (IBM Corporation, Armonk, NY).

## Results

### Part 1: Modulation of DPP‐IV in cell culture using exercise‐related signaling pathways, leucine and whey protein

**Figure 1:**
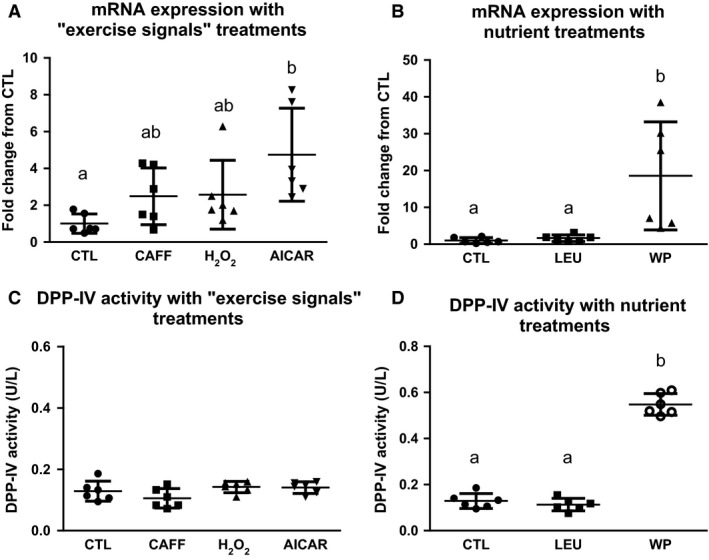
DPP‐IV mRNA expression and activity in cell culture with exercise signals and nutrient treatments. (A) The mRNA expression of DPP‐IV was significantly elevated from that of the control cell culture treatment (CTL) when treated with AICAR (*P* < 0.05), but not with Caffeine (CAFF) or hydrogen peroxide (H2O2). (B) The mRNA expression of DPP‐IV was significantly elevated from that of the CTL cell culture treatment only when treated with Whey Protein (WP;* P *< 0.05), but not Leucine (LEU). (C) DPP‐IV activity of the cell culture media of the “exercise signal” treated cells (H_2_O_2_, CAFF, AICAR) was not significantly different from that of the CTL condition. (D) DPP‐IV activity of the cell culture media was significantly increased when the cells were treated with WP (*P* < 0.05), but not LEU. Conditions sharing the same letter are not significantly different, and those with different letters are significantly different (*P* < 0.05).

Compared to CTL‐treated myotubes, caffeine and H_2_O_2_ treatments increased DPP‐IV mRNA expression by 2.5‐ and 2.6‐fold, but these increases were not significant. However, AICAR significantly increased DPP‐IV mRNA expression 4.75‐fold (*P* = 0.0052) (Fig. 1A). Remarkably, DPP‐IV mRNA expression was significantly increased 18.6‐fold (*P* = 0.0151) in myotubes treated with whey protein when compared to CTL‐treated myotubes, whereas leucine only increased DPP‐IV mRNA expression 1.6‐fold, which was not significant (Fig. 1B). When the release of DPP‐IV into the cell culture media was examined, the activity was significantly increased only with the whey protein treatment (*P* < 0.0001) (Fig. 1C and D).

**Figure 2:**
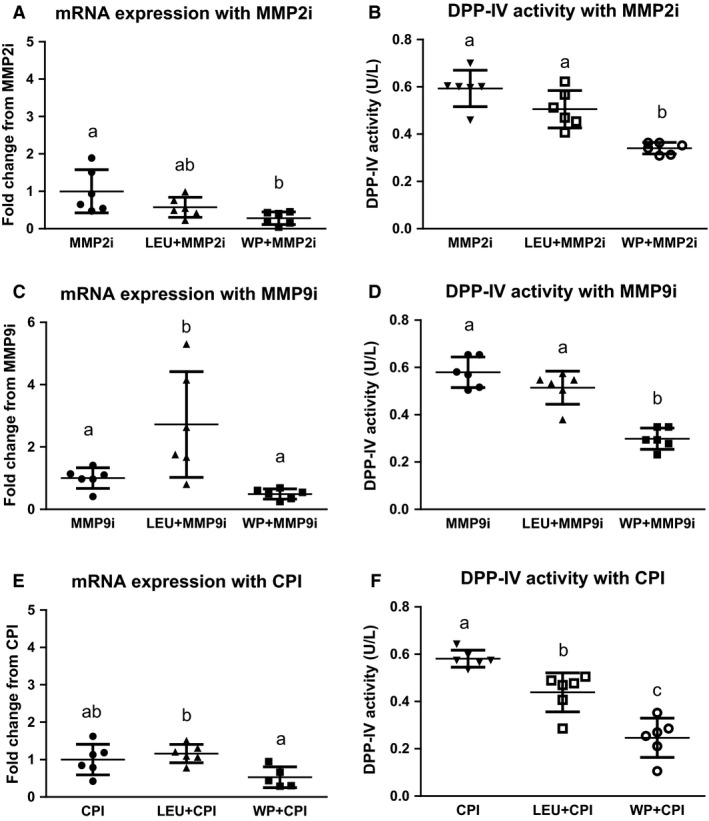
DPP‐IV mRNA expression and activity of cell culture with metalloprotease inhibitors. (A) The mRNA expression of DPP‐IV was significantly decreased by the application of the MMP2 inhibitor (MMP2i) in cell culture only when treated in combination with Whey protein (WP + MMP2i; *P* < 0.05). (B) DPP‐IV activity of the cell culture media was significantly decreased when the cells were treated with WP + MMP2i (*P *< 0.05), but not Leucine (LEU)  + MMP2i. Also, the WP + MMP2i treated cell culture media had significantly lower DPP‐IV activity than LEU + MMP2i (*P* < 0.05) (C) The mRNA expression of DPP‐IV was significantly increased from that of the MMP9 inhibitor treatment (MMP9i) only when treated in combination with LEU (LEU + MMP9i; *P* < 0.05). But the DPP‐IV mRNA expression was significantly lower in the WP + MMP9i cells than that of the LEU + MMP9i cells (*P *< 0.05). (D) DPP‐IV activity of the cell culture media was significantly decreased when the cells were treated with WP + MMP9i (*P* < 0.05), but not LEU + MMP9i. Also, the WP + MMP9i treated cell culture media had significantly lower DPP‐IV activity than LEU + MMP9i (*P* < 0.05) (E) The mRNA expression of DPP‐IV was not significantly changed from that of the Complete Protease Inhibitor treatment (CPI) when treated in combination with Whey protein (WP + CPI) or Leucine and Complete Protease Inhibitor (LEU + CPI). But the DPP‐IV mRNA expression of WP + CPI was significantly lower than that of LEU + CPI (*P* < 0.05). (F) DPP‐IV activity was significantly decreased from that of the Complete Protease Inhibitor control treatment (CPI) when treated with either Whey protein in combination with Complete Protease inhibitor (WP + CPI) or Leucine and Complete Protease Inhibitor (LEU + CPI). Also the DPP‐IV activity of WP + CPI was significantly lower than that of LEU + CPI (*P* < 0.05). Conditions sharing the same letter are not significantly different, and those with different letters are significantly different (*P *< 0.05).

The results of the post hoc cell culture sets showed a significant decrease in DPP‐IV gene expression when cells were treated with MMP2i + WP (*P* = 0.0150), but not MMP2i + LEU (Fig. 2A). Similar results were found in the DPP‐IV activity of the cell culture media. The DPP‐IV activity measured in the media of WP + MMP2i cells was significantly lower than the activity in both MMP2i alone (*P* < 0.0001) and LEU + MMP2i cell media (*P* = 0.0006; Fig. 2B). Additionally, when cells were treated with LEU + MMP9i, mRNA was significantly increased 2.72‐fold (*P* = 0.0351), but decreased with WP + MMP9i, though this decrease was not significant (Fig. 2C). Similar to MMP2i, only WP + MMP9i cells had significantly less DPP‐IV activity in the cell culture media compared to MMP9i alone (*P* < 0.0001) and LEU + MMP9i (*P* < 0.0001; Fig. 2D). In the presence of CPI, neither LEU nor WP caused a significant change in DPP‐IV gene expression; however, the mRNA levels with WP were significantly lower than those with LEU (*P* = 0.0031; Fig. 2E). In the cell culture media, DPP‐IV activity in both WP + CPI and LEU + CPI cells were significantly less than cells treated with CPI alone (*P* < 0.0001 and *P* = 0.0030), with WP + CPI also being significantly less than LEU + CPI (*P* = 0.0024; Fig. 2F).

### Part 2: Modulation of DPP‐IV mRNA and enzyme activity in rats following acute exercise with or without leucine and whey protein feedings

**Figure 3:**
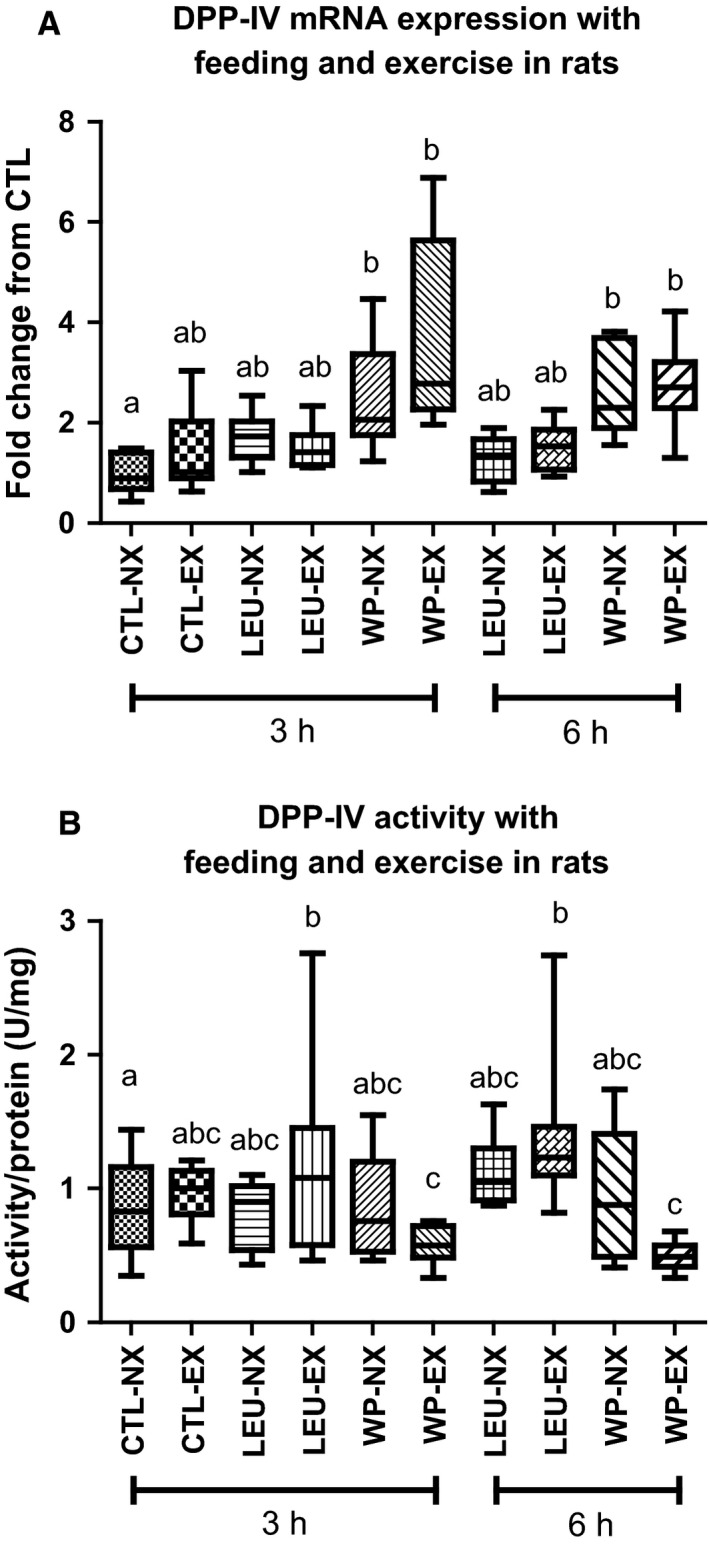
DPP‐IV mRNA expression and activity with feeding and exercise in rats. (A) Only rats fed Whey Protein (NX‐WP and EX‐WP) had significantly increased DPP‐IV mRNA expression compared to the rats fed water (CTL) and leucine (LEU). Time and exercise did not have a significant effect on the mRNA expression of DPP‐IV in any of the feeding treatments. *Denotes statistically different from the control unfed, nonexercised rats (NX‐CTL). (B) DPP‐IV activity was significantly decreased in the exercised gastrocnemius of rats fed Whey Protein (EX‐WP) compared to rats fed water (Water) or leucine (LEU). Conditions sharing the same letter are not significantly different, and those with different letters are significantly different (*P* < 0.05).

The effects of the acute exercise with or without whey protein and leucine feeding following exercise are presented in Figure 3. Only whey protein supplementation significantly increased DPP‐IV gene expression, with no effect of either time or exercise. In the nonexercised gastrocnemius muscles, whey protein alone increased DPP‐IV mRNA expression 2.5‐fold by 3 h post feeding (*P* = 0.01) and DPP‐IV mRNA remained elevated 6 h following feeding (*P* = 0.001). Remarkably, in the exercised gastrocnemius muscles, whey protein feeding increased DPP‐IV mRNA 3.7‐fold 3 h following exercise/feeding (*P* = 0.01), and this remained 2.7‐fold elevated over the CTL condition 6 h following exercise/feeding (*P* = 0.01).

There was no significant effect of time on the enzyme activity of DPP‐IV. However, there was a significant interaction of supplement and exercise (*P* = 0.003). Whey protein supplementation significantly decreased the DPP‐IV activity of the exercised gastrocnemius (3 h = 0.58 ± 0.14 U/mg and 6 h = 0.50 ± 0.11 U/mg), but not the nonexercised muscle when compared with  exercised CTL (0.97 ± 0.20 U/mg; *P* = 0.05) and LEU condition (3 h = 1.18 ± 0.78 U/mg and 6 h = 1.37 ± 0.56 U/mg; *P* = 0.001).

### Part 3: Modulation of serum DPP‐IV activity in humans with whey protein feeding or different types of exercise

**Figure 4:**
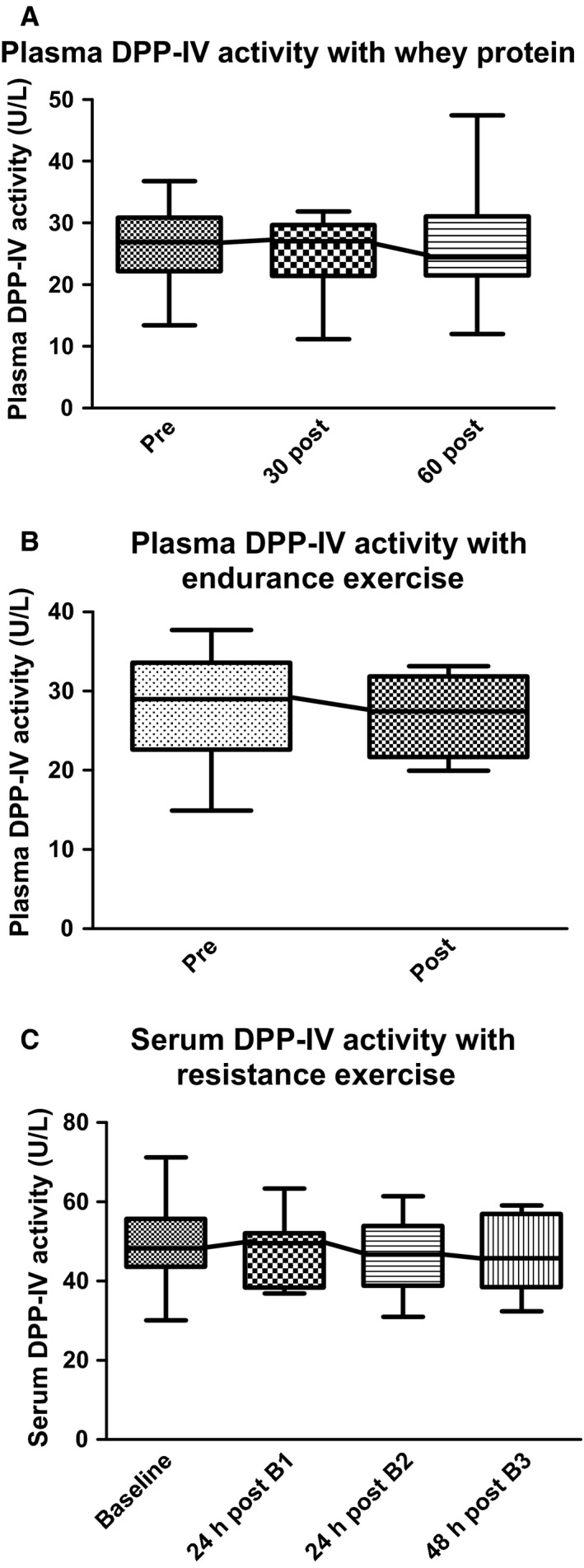
Plasma and serum DPP‐IV activity with feeding and exercise in humans. (A) The plasma DPP‐IV activity was not significantly different 30 and 60 min after ingestion of Whey Protein. (B) The plasma DPP‐IV activity was not significantly different after a vigorous bout of running. (C) Serum DPP‐IV activity was not significantly different following three bouts of resistance exercise.

When participants were fed the whey protein shake, there were no significant differences in the plasma DPP‐IV activity values immediately prior to consumption (26.31 ± 6.86 U/L) versus 30 min (25.49 ± 5.7 U/L) or 60 min (26.13 ± 8.56 U/L) post consumption (Fig. 4A). After a bout of vigorous running, participants presented no significant change in plasma DPP‐IV values 10 min after the cessation of exercise (Pre: 27.74 ± 6.75 U/L and Post: 27.04 ± 4.87 U/L) (Fig. 4B).

Participants completing the 3‐day consecutive resistance training protocol showed no significant difference in serum DPP‐IV activity from baseline (49.14 ± 11.32 U/L) to 24 h post bout 1 (47.28 ± 7.72 U/L), 24 h post bout 2 (46.89 ± 9.72 U/L), or 48 h post bout 3 (46.62 ± 9.72 U/L) (Fig. 4C). There was a decrease in total lifting volume from the first day (2407 ± 401 kg) to the second day (2308 ± 521, *P* = 0.10), but there was a decrease from the first to third day (2285 ± 477, *P* < 0.05) (*data not shown*). Moreover, the visual analog soreness scale (0 = not sore at all, 100 = extremely sore) increased from prior to the first day of lifting (score = 3 ± 6) to 48 h after the third exercise bout (score = 21 ± 12, *P* < 0.001) (*data not shown*).

## Discussion

The purpose of this study was to identify whether nutrient and/or exercise signals affected DPP‐IV production and/or secretion by cultured skeletal muscle cells in vitro and animals and humans in vivo models. In the cell culture experiments, “exercise‐like” modulators (i.e., caffeine, AICAR, and H_2_O_2_) all modestly increased DPP‐IV mRNA expression, but failed to cause release of DPP‐IV into the culture media. However, whey protein resulted in both a robust increase in DPP‐IV mRNA and DPP‐IV activity in the culture media which was in contrast to our proposed hypothesis based on previous literature. This finding was supported in our rat experiments which demonstrated that the largest increase in DPP‐IV mRNA and decrease in intracellular DPP‐IV activity occurred in animals fed whey protein. We contend, however, that the effects of whey protein and exercise on DPP‐IV production and secretion is likely a local effect because when humans were provided with whey protein or acutely exercised, there was no change in plasma DPP‐IV activity. Overall, these data suggest that DPP‐IV transcription may occur through many exercise‐related pathways, but only whey protein and muscle contraction cause DPP‐IV to be released from the muscle. In addition, DPP‐IV release from the muscle does not appear to cause a significant change in the plasma soluble pool of DPP‐IV activity.

### Part 1: Modulation of DPP‐IV in cell culture using exercise‐related signaling pathways, amino acids and whey protein

Myotubes were treated with the “exercise‐like” mimetics, caffeine (to increase cytosolic Ca^2+^), AICAR (for AMPK activation), and H_2_O_2_ (for redox signaling). When exercise‐like signals were evoked, we anticipated that DPP‐IV mRNA expression and release would be increased based on recent research (Raschke et al. 2013). This was supported when all of the “exercise‐like” mimetics caused an increase in the mRNA expression of DPP‐IV, with AICAR having the greatest effect. However, when DPP‐IV activity was measured in the media, the “exercise‐like” mimetics did not significantly change the release of DPP‐IV compared to the control condition. As intracellular DPP‐IV activity was not measured, it remains unknown if this increase in DPP‐IV mRNA was translated into protein within the muscle cell. To date, there are no known intracellular pathways in which DPP‐IV is directly involved, as DPP‐IV has been primarily identified as an extracellular‐agent (Kirby et al. 2010). It is possible that there was an increase in membrane‐bound DPP‐IV following the exercise signals, but our experiments did not provide a mechanism or enough time to cause DPP‐IV to be released into the media.

When hydrolyzed whey protein or the single amino acid, leucine, was applied to the myotubes, an increase was found in DPP‐IV mRNA expression in the cells, with whey protein providing a more robust effect. Since leucine is a component of whey protein that increases mTOR activation, these data suggest that leucine and/or mTOR activation do not play an important role in stimulating transcription of DPP‐IV. When DPP‐IV activity was measured in the media, leucine did not significantly change the release of DPP‐IV, whereas whey protein resulted in a significant increase in DPP‐IV activity in the culture media. The findings of this study were further supported by cell culture data showing that inhibition of metalloproteases 2 and 9 in the presence of whey protein caused a decrease in the mRNA expression and media activity of DPP‐IV. MMP 2 and 9, which are involved in the shedding of membrane‐bound DPP‐IV (Röhrborn et al. 2014), have been reported as a component of whey protein (Raulo et al. 2002; Lubetzky et al. 2010) providing a mechanism for the increase in the DPP‐IV found in the media after myotubes were exposed to whey protein.

These findings were surprising considering that previous studies demonstrated that whey protein inhibits DPP‐IV. There are several possible explanations for this contrast in findings. One possibility may be that ingested whey protein has a different effect in the gut than it does in the muscle. In studies investigating whey protein as an inhibitor of DPP‐IV using in vitro methodology, inhibition was only measured up to 45 min post treatment (Tulipano et al. 2011; Silveira et al. 2013). In this study, whey protein activated a pathway specific to muscle that produces more DPP‐IV mRNA up to 6 h after the initial treatment. In addition, other studies measured inhibition in a solution containing only recombinant DPP‐IV (Tulipano et al. 2011; Silveira et al. 2013), whereas this study examined DPP‐IV production in metabolically active muscle cells. The bioactive component in whey protein that is eliciting increases in DPP‐IV mRNA expression and activity remains elusive. However, others have studied the effects of whey protein bioactives on various facets of physiology. For instance, Gaudel et al. reported that treating pancreatic cells in culture with hydrolyzed whey protein elicited a robust increase in insulin secretion (Gaudel et al. 2013). Others have reported that hydrolyzed whey protein ingestion reduces circulating GLP‐1 in humans (Aziz et al. 2005), an effect that may be mediated by bioactive peptides from hydrolyzed whey protein. Finally, a recent review article notes that milk‐derived whey contains bioactive peptides which have been shown to elicit opioid agonistic effects as well as angiotensinogen‐converting enzyme inhibition (Raikos and Dassios 2013). Thus, hydrolyzed whey protein may possess bioactive peptides that could act to modulate DPP‐IV expression and activity; this needs to be researched in further detail.

Another interesting finding resulted when the cultured cells were exposed to leucine and MMP9i; DPP‐IV mRNA increased, but there was no change in DPP‐IV release into the media. The reason for this increase in mRNA remains unknown. The original rationale for including leucine in the study was to test the hypothesis that amino acid transport was the primary signal for DPP‐IV release. However, this supposition was disproved when leucine failed to cause release of DPP‐IV in cell culture. Nevertheless, we posit that metalloproteases in whey protein may cause DPP‐IV shedding from myotubes as metalloproteases have been reported in the past to elicit this effect. More research is needed to uncover the mechanism for DPP‐IV mRNA increases in the presence of LEU + MMP9i.

### Part 2: Modulation of DPP‐IV in animal model with muscle stimulation, amino acid feeding, and whey protein feeding

In order to test if the effect observed in cell culture occurred in vivo, we chose to employ a rat model in which their right gastrocnemius was acutely exercised with or without leucine or whey protein feedings following exercise. In agreement with our cell culture data, postexercise whey protein feeding resulted in a significant increase in DPP‐IV mRNA at both 3 and 6 h post exercise, while muscle stimulation alone did not cause a significant increase in DPP‐IV mRNA. However, when muscle stimulation was combined with subsequent whey feeding, there appeared to be a slight additive effect, resulting in a larger increase in DPP‐IV mRNA at the 3 h post exercise time point compared to the whey protein fed only muscle, though they were not statistically different. By 6 h post exercise, the DPP‐IV mRNA levels of the exercise plus whey protein muscles were similar to that of the whey protein feeding only muscles.

Curiously, when the DPP‐IV activity of the muscle was measured, whey protein supplementation resulted in a paradoxical decrease in DPP‐IV activity, whereas leucine caused a nonsignificant increase in DPP‐IV activity. Based on our cell culture findings that show whey protein results in the release of DPP‐IV from the cell membrane, our cell culture and rat data collectively suggest that whey protein may cause an accelerated release of DPP‐IV from the muscle which, in turn, depletes the intracellular pool and initiates a robust increase in DPP‐IV gene expression. However, this conclusion is limited by the lack of evidence of DPP‐IV release from the rat skeletal muscle. Leucine, on the other hand, possibly increases the intracellular activity of DPP‐IV, but likely does not deplete intramuscular pools enough to trigger the transcription of DPP‐IV mRNA. To our knowledge, no studies have used animal models to investigate the effect of dietary proteins, amino acids, and/or exercise on skeletal muscle DPP‐IV production. The only research pertaining to the effect of whey and leucine were demonstrated in assays and gastrointestinal studies in vitro (Tulipano et al. 2011; Lacroix and Li‐Chan 2012; Silveira et al. 2013).

### Part 3: Modulation of DPP‐IV in humans with whey protein feeding or different types of exercise

Studies in humans were also performed to see if DPP‐IV secretion from skeletal muscle could impact the soluble DPP‐IV activity pool in the blood. We found that neither the consumption of whey protein nor completion of vigorous exercise altered whole body plasma DPP‐IV activity. It is well understood that DPP‐IV activity in the blood comes from multiple sources including the T‐cells, the gut, adipocytes, and muscle (Lambeir et al. 2003; Cordero et al. 2009; Raschke et al. 2013), but the relative contribution of each is difficult to determine.

In our repeated bout resistance exercise protocol, which resulted in both increased muscle soreness and muscle damage markers (Mumford et al. 2015), there was no change in serum soluble DPP‐IV activity over the duration of the training intervention and up to 48 h following the last training bout. Moreover, a single bout of endurance exercise did not alter the plasma levels of DPP‐IV. This suggests that if human muscle cells release DPP‐IV, it is likely a local phenomenon that cannot be detected through venous blood sampling. It is possible that the resistance and the treadmill exercise bouts caused an increase in CD26+ cells, but the assay is only sensitive to soluble DPP‐IV (Yu et al. 2011). Additionally, a limitation of the treadmill study was that plasma DPP‐IV was only measured 10 min post exercise, and there may be a further time point that should be examined with endurance exercise. Therefore, further studies should investigate the response of inflammatory CD26+ T cells to exercise.

The gastrointestinal (GI) system is also a source of DPP‐IV, with the highest concentration in the body occurring along the intestinal wall (Lambeir et al. 2003). Moreover, the GI system plays a major role in regulating the amount of active GLP‐1 in the plasma, especially after the consumption of a meal (D'Alessio 2011; De Silva and Bloom 2012). Previous work suggests that whey protein acts as a DPP‐IV inhibitor (Tulipano et al. 2011; Lacroix and Li‐Chan 2012), implying that with its ingestion, the amount of DPP‐IV activity in the plasma would decrease. This was not found in this study, as there was no significant change in DPP‐IV activity at 30 or 60 min following whey protein ingestion. It is possible that whey protein acts as a DPP‐IV inhibitor in the gut postprandium, but this inhibition is not large enough to cause a change in soluble plasma DPP‐IV activity, at least with the amount of protein consumed for this study.

DPP‐IV has been reported to be a myokine in other cell culture studies (Raschke et al. 2013). However, it remains unknown if DPP‐IV released by the muscle can travel to the blood and contribute to the soluble DPP‐IV pool in the blood. Our data suggest that if DPP‐IV is released by human skeletal muscle in response to whey protein ingestion, it does not contribute to the soluble pool of DPP‐IV in the blood. In this regard, whey protein‐induced increases in DPP‐IV originating from the muscle could remain in the interstitial space and elicit local paracrine effects, but this effect is not large enough to cause a whole body change in circulating levels.

In conclusion, we report that: (1) exercise‐like mimetics in vitro modestly increase DPP‐IV mRNA, but this does not translate to secretion and, (2) whey protein robustly increases DPP‐IV mRNA although this does translate to secretion, (3) whey protein increases DPP‐IV mRNA postexercise in vivo in rodents, regardless of time and exercise, (4) whey protein feeding alone does not affect DPP‐IV activity in the plasma in humans in vivo, (5) acute endurance exercise does not affect plasma DPP‐IV activity up to 10 min post exercise in humans in vivo, and (6) three repeated bouts of resistance training does not affect serum DPP‐IV activity in humans in vivo up to 48 h following the last bout. Collectively, our cell culture and rat data suggest DPP‐IV is being produced by skeletal muscle, and our in vitro data suggest that the DPP‐IV myokine is being released by skeletal muscle into the interstitial space. However, DPP‐IV function outside of the muscle is inconclusive based on the lack of local measures of DPP‐IV in both the rat and human studies. However, the human data suggest that if DPP‐IV is released by the skeletal muscle, it is not upregulated in circulation and likely exhibits autocrine or paracrine effects. Further research should investigate the local effects of DPP‐IV from the muscle with different stimuli. Also, specific pathways should be studied with techniques to measure local DPP‐IV. These findings could expand the role of DPP‐IV from whole body energy metabolism (via incretin hormones, neuropeptide Y, and PYY) to modulation of muscle function. Some possible areas in which DPP‐IV could be involved are the modulation of blood flow via vasoactive NPY (Evanson et al. 2011) and satellite cell proliferation through interactions with SDF‐1 (Kucia et al. 2004) and IL‐6 (Hoffmann et al. 1993; Serrano et al. 2008; Toth et al. 2011).

## Conflict of Interest

None declared.

## References

[phy212827-bib-0001] Aziz, A. , G. H. Anderson , A. Giacca , and F. Cho . 2005 Hyperglycemia after protein ingestion concurrent with injection of a GLP‐1 receptor agonist in rats: a possible role for dietary peptides. Am. J. Physiol. 289:R688–R694. 10.1152/ajpregu.00850.2004 15879053

[phy212827-bib-0002] Badman, M. K. , and J. S. Flier . 2005 The gut and energy balance: visceral allies in the obesity wars. Science 307:1909–1914. 15790843 10.1126/science.1109951

[phy212827-bib-0003] Barres, R. , J. Yan , B. Egan , J. T. Treebak , M. Rasmussen , T. Fritz , et al. 2012 Acute exercise remodels promoter methylation in human skeletal muscle. Cell Metab. 15:405–411. 22405075 10.1016/j.cmet.2012.01.001

[phy212827-bib-0004] Batterham, R. L. , H. Heffron , S. Kapoor , J. E. Chivers , K. Chandarana , H. Herzog , et al. 2006 Critical role for peptide YY in protein‐mediated satiation and body‐weight regulation. Cell Metab. 4:223–233. 16950139 10.1016/j.cmet.2006.08.001

[phy212827-bib-0005] Beaton, L. J. , M. A. Tarnopolsky , and S. M. Phillips . 2002 Contraction‐induced muscle damage in humans following calcium channel blocker administration. J. Physiol. 544:849–859. 12411528 10.1113/jphysiol.2002.022350 PMC2290613

[phy212827-bib-0006] Butteiger, D. , M. Cope , P. Liu , R. Mukherjea , E. Volpi , B. Rasmussen , et al. 2013 A soy, whey and caseinate blend extends postprandial skeletal muscle protein synthesis in rats. Clin. Nutr. 32:585–591. 23127543 10.1016/j.clnu.2012.10.001 PMC4164044

[phy212827-bib-0007] Cordero, O. J. , F. J. Salgado , and M. Nogueira . 2009 On the origin of serum CD26 and its altered concentration in cancer patients. Cancer Immunol. Immunother. 58:1723–1747. 19557413 10.1007/s00262-009-0728-1 PMC11031058

[phy212827-bib-0008] D'Alessio, D. A. 2011 What if gut hormones aren't really hormones: DPP‐4 inhibition and local action of GLP‐1 in the gastrointestinal tract. Endocrinology 152:2925. 21785013 10.1210/en.2011-1385 PMC3138230

[phy212827-bib-0009] De Silva, A. , and S. R. Bloom . 2012 Gut hormones and appetite control: a focus on PYY and GLP‐1 as therapeutic targets in obesity. Gut. Liv. 6:10–20. 10.5009/gnl.2012.6.1.10 PMC3286726 22375166

[phy212827-bib-0010] DeLong, A. D. , R. Pratt , and T. J. McLoughlin . 2011 FoxO1‐mediated disruption of the Akt/mTOR signaling pathway in skeletal myotubes. FASEB J. 25:lb603.

[phy212827-bib-0011] Dourado, M. , A. B. Sarmento , S. V. Pereira , V. Alves , T. Silva , A. M. Pinto , et al. 2007 CD26/DPPIV expression and 8‐azaguanine response in T‐acute lymphoblastic leukaemia cell lines in culture. Pathophysiology 14:3–10. 17055708 10.1016/j.pathophys.2006.09.003

[phy212827-bib-0012] Dreyer, H. C. , S. Fujita , J. G. Cadenas , D. L. Chinkes , E. Volpi , and B. B. Rasmussen . 2006 Resistance exercise increases AMPK activity and reduces 4E‐BP1 phosphorylation and protein synthesis in human skeletal muscle. J. Physiol. 576:613–624. 16873412 10.1113/jphysiol.2006.113175 PMC1890364

[phy212827-bib-0013] Egawa, T. , Y. Ohno , A. Goto , A. Ikuta , M. Suzuki , T. Ohira , et al. 2014 AICAR‐induced activation of AMPK negatively regulates myotube hypertrophy through the HSP72‐mediated pathway in C2C12 skeletal muscle cells. Am. J. Physiol. Endocrinol. Metabol. 306:E344–E354. 10.1152/ajpendo.00495.2013 24347059

[phy212827-bib-0014] Evanson, K. W. , A. J. Stone , A. L. Hammond , and H. A. Kluess . 2011 Neuropeptide Y overflow and metabolism in skeletal muscle arterioles. J. Physiol. 589:3309–3318. 21558160 10.1113/jphysiol.2011.209726 PMC3145941

[phy212827-bib-0015] Frid, A. H. , M. Nilsson , J. J. Holst , and I. M. Björck . 2005 Effect of whey on blood glucose and insulin responses to composite breakfast and lunch meals in type 2 diabetic subjects. Am. J. Clin. Nutr. 82:69–75. 16002802 10.1093/ajcn.82.1.69

[phy212827-bib-0016] Gaudel, C. , A. B. Nongonierma , S. Maher , S. Flynn , M. Krause , B. A. Murray , et al. 2013 A whey protein hydrolysate promotes insulinotropic activity in a clonal pancreatic *β*‐cell line and enhances glycemic function in ob/ob mice. J. Nutr. 143:1109–1114. 23658425 10.3945/jn.113.174912

[phy212827-bib-0017] Hoffmann, T. , J. Faust , K. Neubert , and S. Ansorge . 1993 Dipeptidyl peptidase IV (CD 26) and aminopeptidase N (CD 13) catalyzed hydrolysis of cytokines and peptides with N‐terminal cytokine sequences. FEBS Lett. 336:61–64. 7903256 10.1016/0014-5793(93)81609-4

[phy212827-bib-0018] Hulmi, J. J. , C. M. Lockwood , and J. R. Stout . 2010 Review effect of protein/essential amino acids and resistance training on skeletal muscle hypertrophy: a case for whey protein. Nutr. Metab. 7:51. 10.1186/1743-7075-7-51 PMC2901380 20565767

[phy212827-bib-0019] Ikeda, T. , E. Kumagai , S. Iwata , and A. Yamakawa . 2013 Soluble CD26/dipeptidyl peptidase IV enhances the transcription of IL‐6 and TNF‐*α* in THP‐1 cells and monocytes. PLoS ONE 8:e66520. 23805228 10.1371/journal.pone.0066520 PMC3689814

[phy212827-bib-0020] Kirby, M. , D. Yu , S. O'Connor , and M. Gorrell . 2010 Inhibitor selectivity in the clinical application of dipeptidyl peptidase‐4 inhibition. Clin. Sci. 118:31–41. 19780719 10.1042/CS20090047

[phy212827-bib-0021] Kucia, M. , K. Jankowski , R. Reca , M. Wysoczynski , L. Bandura , D. J. Allendorf , et al. 2004 CXCR4–SDF‐1 signalling, locomotion, chemotaxis and adhesion. J. Mol. Histol. 35:233–245. 15339043 10.1023/b:hijo.0000032355.66152.b8

[phy212827-bib-0022] Lacroix, I. M. , and E. C. Li‐Chan . 2012 Dipeptidyl peptidase‐IV inhibitory activity of dairy protein hydrolysates. Int. Dairy J. 25:97–102.

[phy212827-bib-0023] Lambeir, A.‐M. , C. Durinx , S. Scharpé , and I. De Meester . 2003 Dipeptidyl‐peptidase IV from bench to bedside: an update on structural properties, functions, and clinical aspects of the enzyme DPP IV. Crit. Rev. Clin. Lab. Sci. 40:209–294. 12892317 10.1080/713609354

[phy212827-bib-0024] Lamers, D. , S. Famulla , N. Wronkowitz , S. Hartwig , S. Lehr , D. M. Ouwens , et al. 2011 Dipeptidyl peptidase 4 is a novel adipokine potentially linking obesity to the metabolic syndrome. Diabetes 60:1917–1925. 21593202 10.2337/db10-1707 PMC3121429

[phy212827-bib-0025] Loh, K. , H. Herzog , and Y.‐C. Shi . 2015 Regulation of energy homeostasis by the NPY system. Trends Endocrinol. Metab. 26:125–135. 25662369 10.1016/j.tem.2015.01.003

[phy212827-bib-0026] Lubetzky, R. , D. Mandel , F. B. Mimouni , L. Herman , R. Reich , and S. Reif . 2010 MMP‐2 and MMP‐9 and their tissue inhibitor in preterm human milk. J. Pediatr. Gastroenterol. Nutr. 51:210–212. 20531021 10.1097/MPG.0b013e3181d345b8

[phy212827-bib-0027] Matheeussen, V. , A.‐M. Lambeir , W. Jungraithmayr , N. Gomez , K. Mc Entee , P. Van der Veken , et al. 2012 Method comparison of dipeptidyl peptidase IV activity assays and their application in biological samples containing reversible inhibitors. Clin. Chim. Acta 413:456–462. 22093941 10.1016/j.cca.2011.10.031

[phy212827-bib-0028] McClung, J. M. , D. Van Gammeren , M. A. Whidden , D. J. Falk , A. N. Kavazis , M. B. Hudson , et al. 2009 Apocynin attenuates diaphragm oxidative stress and protease activation during prolonged mechanical ventilation. Crit. Care Med. 37:1373. 19242334 10.1097/CCM.0b013e31819cef63 PMC2909189

[phy212827-bib-0029] McGinnis, G. R. , C. Ballmann , B. Peters , G. Nanayakkara , M. Roberts , R. Amin , et al. 2015 Interleukin‐6 mediates exercise preconditioning against myocardial ischemia reperfusion injury. Am. J. Physiol. Heart Circ. Physiol. 308:H1423–H1433. 25820396 10.1152/ajpheart.00850.2014

[phy212827-bib-0030] Mobley, C. B. , C. D. Fox , B. S. Ferguson , C. A. Pascoe , J. C. Healy , J. S. McAdam , et al. 2015 Effects of protein type and composition on postprandial markers of skeletal muscle anabolism, adipose tissue lipolysis, and hypothalamic gene expression. J. Int. Soc. Sports Nutr. 12:14. 25792976 10.1186/s12970-015-0076-9 PMC4365970

[phy212827-bib-0031] Mobley, C. B. , C. D. Fox , R. M. Thompson , J. C. Healy , V. Santucci , W. C. Kephart , et al. 2016 Comparative effects of whey protein versus l‐leucine on skeletal muscle protein synthesis and markers of ribosome biogenesis following resistance exercise. Amino Acids 48:733–750. 26507545 10.1007/s00726-015-2121-z

[phy212827-bib-0032] Mumford, P. , W. C. Kephart , A. E. McCloskey , A. M. Holland , J. J. Shake , C. B. Mobley , et al. 2015 Effects of sub‐chronic branched chain amino acid supplementation on markers of muscle damage and performance variables following 1 week of rigorous weight training. J. Int. Soc. Sports Nutr. 12:P29.

[phy212827-bib-0033] Raikos, V. , and T. Dassios . 2013 Health‐promoting properties of bioactive peptides derived from milk proteins in infant food: a review. Dairy Sci. Technol. 94:91–101. 24511365 10.1007/s13594-013-0152-3 PMC3912356

[phy212827-bib-0034] Raschke, S. , K. Eckardt , K. B. Holven , J. Jensen , and J. Eckel . 2013 Identification and validation of novel contraction‐regulated myokines released from primary human skeletal muscle cells. PLoS ONE 8:e62008. 23637948 10.1371/journal.pone.0062008 PMC3634789

[phy212827-bib-0035] Raulo, S. M. , T. Sorsa , T. Tervahartiala , T. Latvanen , E. Pirilä , J. Hirvonen , et al. 2002 Increase in milk metalloproteinase activity and vascular permeability in bovine endotoxin‐induced and naturally occurring Escherichia coli mastitis. Vet. Immunol. Immunopathol. 85:137–145. 11943315 10.1016/s0165-2427(01)00423-8

[phy212827-bib-0036] Reagan‐Shaw, S. , M. Nihal , and N. Ahmad . 2008 Dose translation from animal to human studies revisited. FASEB J. 22:659–661. 17942826 10.1096/fj.07-9574LSF

[phy212827-bib-0037] Reid, M. B. , and W. J. Durham . 2002 Generation of reactive oxygen and nitrogen species in contracting skeletal muscle. Ann. N. Y. Acad. Sci. 959:108–116. 11976190 10.1111/j.1749-6632.2002.tb02087.x

[phy212827-bib-0038] Reidy, P. T. , D. K. Walker , J. M. Dickinson , D. M. Gundermann , M. J. Drummond , K. L. Timmerman , et al. 2013 Protein blend ingestion following resistance exercise promotes human muscle protein synthesis. J. Nutr. 143:410–416. 23343671 10.3945/jn.112.168021 PMC3738242

[phy212827-bib-0039] Röhrborn, D. , J. Eckel , and H. Sell . 2014 Shedding of dipeptidyl peptidase 4 is mediated by metalloproteases and up‐regulated by hypoxia in human adipocytes and smooth muscle cells. FEBS Lett. 588:3870–3877. 25217834 10.1016/j.febslet.2014.08.029

[phy212827-bib-0040] Salehi, A. , U. Gunnerud , S. J. Muhammed , E. Ostman , J. J. Holst , I. Björck , et al. 2012 The insulinogenic effect of whey protein is partially mediated by a direct effect of amino acids and GIP on beta‐cells. Nutr. Metab. (Lond) 9:48. 22647249 10.1186/1743-7075-9-48 PMC3471010

[phy212827-bib-0041] Scharpé, S. , I. De Meester , G. Vanhoof , D. Hendriks , M. Van Sande , K. Van Camp , et al. 1988 Assay of dipeptidyl peptidase IV in serum by fluorometry of 4‐methoxy‐2‐naphthylamine. Clin. Chem. 34:2299–2301. 2902942

[phy212827-bib-0042] Serrano, A. L. , B. Baeza‐Raja , E. Perdiguero , M. Jardí , and P. Muñoz‐Cánoves . 2008 Interleukin‐6 is an essential regulator of satellite cell‐mediated skeletal muscle hypertrophy. Cell Metab. 7:33–44. 18177723 10.1016/j.cmet.2007.11.011

[phy212827-bib-0043] Silveira, S. T. , D. Martínez‐Maqueda , I. Recio , and B. Hernández‐Ledesma . 2013 Dipeptidyl peptidase‐IV inhibitory peptides generated by tryptic hydrolysis of a whey protein concentrate rich in *β*‐lactoglobulin. Food Chem. 141:1072–1077. 23790888 10.1016/j.foodchem.2013.03.056

[phy212827-bib-0044] Toth, K. G. , B. R. McKay , M. De Lisio , J. P. Little , M. A. Tarnopolsky , and G. Parise . 2011 IL‐6 induced STAT3 signalling is associated with the proliferation of human muscle satellite cells following acute muscle damage. PLoS ONE 6:e17392–e17392. 21408055 10.1371/journal.pone.0017392 PMC3052298

[phy212827-bib-0045] Tulipano, G. , V. Sibilia , A. M. Caroli , and D. Cocchi . 2011 Whey proteins as source of dipeptidyl dipeptidase IV (dipeptidyl peptidase‐4) inhibitors. Peptides 32:835–838. 21256171 10.1016/j.peptides.2011.01.002

[phy212827-bib-0046] Yu, D. , L. Slaitini , V. Gysbers , A. Riekhoff , T. Kähne , H. Knott , et al. 2011 Soluble CD26/dipeptidyl peptidase IV enhances human lymphocyte proliferation in vitro independent of dipeptidyl peptidase enzyme activity and adenosine deaminase binding. Scand. J. Immunol. 73:102–111. 21198750 10.1111/j.1365-3083.2010.02488.x

[phy212827-bib-0047] Zhang, D. , W. Huang , B. Dai , T. Zhao , A. Ashraf , R. W. Millard , et al. 2010 Genetically manipulated progenitor cell sheet with diprotin A improves myocardial function and repair of infarcted hearts. Am. J. Physiol. Heart Circ. Physiol. 299:H1339–H1347. 20802132 10.1152/ajpheart.00592.2010 PMC2993193

